# Clinical Features of 33 Cases in Children Infected With SARS-CoV-2 in Anhui Province, China–A Multi-Center Retrospective Cohort Study

**DOI:** 10.3389/fpubh.2020.00255

**Published:** 2020-06-16

**Authors:** Lan Zhang, Songming Huang

**Affiliations:** Pediatric Department Fourth Affiliated Hospital of Nanjing Medical University, Nanjing, China

**Keywords:** COVID-19, SARS-CoV-2, children, chest CT, susceptibility, familial clustering

## Abstract

**Background:** As of 23rd February 2020, China had 77,048 patients with confirmed SARS-CoV-2 infections, and only 2. 1% of patients were under the age of 19 years. Morbidity among children was much lower, with milder or absent signs and symptoms; chest CT scans showed milder symptoms, if at all, compared to adults.

**Objective:** Report the epidemiological, clinical features, laboratory, radiological characteristics, and treatment of SARS-CoV-2 infections. Compare additional signs and symptoms, investigate familial clustering, compare laboratory results, and find out relevance between age and typical chest CT scans in patients.

**Methods:** We studied 33 young patients with laboratory-confirmed SARS-CoV-2 infection in Anhui Province of China by 16th February 2020. Their signs, symptoms, and familial clustering were analyzed. We compared the laboratory test results, age, and gender among three parts based on their chest CT scans.

**Results:** Familial clustering was seen in 30 (30/33; 90.91%) patients; three families had seven confirmed members infected with the disease. Eight (8/33; 24.24%) patients had no symptoms, 12 (12/33; 36.36%) patients had only fever, nine (9/33; 27.27%) patients had fever and additional symptoms, and 12 (12/33; 36.36%) patients had no fever. Dry cough was the most common additional symptom. In 25 (25/33; 75.76%) patients, the percent of lymphocytes decreased; 26 (26/33; 78.79%) patients were older than 7 years. More male than female patients and patients older than 8 years showed typical abnormalities in the chest CT scans (*P* = 0.038). Only two 18 years old patients had hepatic injury.

**Conclusion:** Children's infection is mild and familial clustering was the most common channel. The older patients had more typical ground glass opacity (GGO) or consolidation in chest CT scans. Cases without fever strongly suggested that non-symptomatic children should not be assumed to be free of infection when their family members have confirmed infection. Most children showed clinical features distinguishable from adults and with increased susceptibility within family members.

## Introduction

Since December 2019, the epidemic of coronavirus−2019 (SARS-COV-2) has spread throughout the world, rapidly resulting in 4,330,982 confirmed cases and 295,671 deaths as of 6th May 2020. Anhui province was the third region to be affected by coronavirus-2019 in China, with Hubei and Guangdong provinces being the first two. Among the patients, only 2.1% were under the age of 19 years. Not only was their morbidity less than adults, their clinical features were also milder. And a few of them showed no signs and symptoms of the infection.

However, every child with a confirmed SARS-COV-2 infection is being diagnosed as having novel coronavirus pneumonia (“NCP”), even though some of them had no fever, cough, fatigue, or typical radiological characteristics in a chest CT.

Here, we report 33 patients under the age of 19 years with confirmed COVID-19 infection from Anhui province, China, and describe the clinical features, laboratory, and radiological characteristics of a chest CT, treatment, and clinical outcome. We also report the patients' history of contact with infected person/s (direct or indirect), and (familial clustering). These cases highlight the importance of familial clustering clinical features, chest CT characteristics, and age. We aim to share our findings and recommend that pediatricians reconsider the diagnoses of children with confirmed infection.

## Patients and Methods

A total of 33 patients were enrolled in this study who were admitted to one of the 10 hospitals in Anhui province in China between December 2019 and February 2020. The inclulsion criteris was: being under 19 years of age having respiratory specimens that were analyzed twice by real-time RT-PCR, and being diagnosed according to the World Health Organization's interim guidance (1). All the cases were discharged with twice negative real-time RT-PCR up to 6th May 2020. All case data can be provided on request.

Thirteen cases from Bozhou People's Hospital, seven cases from No. 2 People's Hospital of Fuyang City, four cases from Wanbei Coal-Electricity Group General Hospital of minors, two cases from The Second People's Hospital of Wuhu, two cases from Anhui Provincial Children's Hospital, one case from The First Affiliated Hospital of USTC, Division of Life Sciences and Medicine, University of Science and Technology of China, one case from Ma'anshan maternal and child health care hospital, one case from The First Affiliated Hospital of Bengbu Medical College, one case from The People's hospital of Lu'an City, and one case from the Maternity and Children Health Care Hospital of Tongling City.

The medical data were analyzed by the medical team from the pediatric department at the First Affiliated Hospital of USTC. Information recorded included demographic data, medical history, familial clustering, details of the confirmed patients, if any, in the family, whether they were residents of Wuhan, or traveled to Wuhan, whether they came in contact with confirmed patients, signs, and symptoms, including pharyngodynia, fever, cough, vomiting and diarrhea, fatigue, tightness in the chest, total WBC and lymphocyte percentages, levels of C-reactive protein (CRP), IL-6, liver function, CKMB, a marker of myocardial injury, chest CT, administration of INF a, lopinavir and ritonavir, ribavirin, or arbidol, and titers of Mp-IgM, anti-parainfluenza virus IgM, anti-influenza virus IgM, and anti-adenovirus IgM. The laboratory test results and statistical analyses were the first ones carried out since the symptoms were noticed.

As lymphocyte population vary according to age a lymphocyte content of <60% in patients below 7 years of age and <30% in patients over 7 years of age is considered as “lymphocyte percentage decrease.”

We divided the chest CT images into three classes: (1) typical abnormalities, with bilateral multiple lobular and subsegmental areas of consolidation or bilateral ground-glass opacity (GGO) and subsegmental areas of consolidation or GGO; (2) non-typical abnormalities, showing nodal and patchy shadow of bilateral median and extrapulmonary zone; and (3) Normal.

We divided the 33 cases under study based on various aspects. When the incidence of fever was considered, they were classified into two groups: with fever (2) and without fever, and the baseline characteristics and differences in other signs and symptoms between the two groups were analyzed. From these data, the percentage of confirmed familial cluster among the cases, and the predominance of different signs and symptoms in the cases were estimated. Based on the laboratory results, we divided the cases into three phases: total WBC ≤ 5X109/L, 5–10X109/L, and >10X109/L, and counted the cases in different phases. We also divided the cases into two categories: decreased and non-decreased, based on the percentage of lymphocytes, and scored the number of cases in each of these categories. When the radiological characteristics of chest CT were considered, the cases were divided into three parts: typical, non- typical, and normal. We also considered differences based on age and gender, details of the treatment, including the drugs administered in all cases, and identified the most widely used ones among these cases.

### Statistics

A retrospective cohort study was used to analyze the epidemiological data, clinical symptoms, and signs, changes in WBC and total lymphocyte counts, chest CT, and the different treatments in children infected with SARS-COV-2. A comparison of the baseline characteristics of the data and signs and symptoms revealed that in both the groups fever was a common symptom. The data were analyzed using cases number (*n*) and percentage (%), except for the age of the patients, which was calculated as the mean. Cases were divided into three categories, according to the severity of chest CT (typical, non-typical, and normal), and compared the differences in age and sex between the three categories. Variables between these were presented as numbers and percentages, and continuous variables were presented as mean ± standard deviation. Chi-square test or Fisher's exact test was used to compare categorical variables, and Student's *t*-test was used for continuous variables. A two-sided *p* < 0.05 was considered statistically significant. Data were analyzed using SPSS Statistics version 19.0 (SPSS Inc., Chicago, IL, USA).

### Ethics

The studies involving human participants were reviewed and approved by Anhui Provincial Hospital (The First Affiliated Hospital of USTC) Medical Research Ethics Committee. Written informed consent to participate in this study was provided by the participants' legal guardian/next of kin. In particular, written informed consent was obtained from the individual(s) for the publication of any potentially identifiable images or data included in this article.

## Results

Among the 33 cases, the fever group (*n* = 21) had more patients than the non-fever group (*n* = 12). Baseline characteristics, including demographic data, familial clusters, Wuhan residence, travel to Wuhan, and contact with confirmed patients were not significantly different between the two groups (Table 1).

**Table 1:** Baseline characteristics of children patients infected with SARS-COV-2.

**No. (%)**	**Total (*n* = 33)**	**Fever (*n* = 21)**	**Non-fever (*n* = 12)**	***t/χ*^**2**^**	^**^p-value
^*^Age	9.59 ± 5.12	11.50 ± 3.93	−1.118		0.272^*^
^#^Sex					
Female	17	11	6	0.017	0.895
Male	16	10	6		
^#^Familial cluster (yes)	30	18 (85.7%)	12 (100%)	1.886	0.284
^#^Wuhan residence (yes)	8	5 (23.3%)	3 (25.0%)	0.006	1.0
^#^Travel in Wuhan (yes)	3	2 (4.3%)	1 (8.3%)	0.265	0.607
^#^Contact confirmed patients (yes)	30	18 (85.7%)	12 (100%)	1.886	0.284

* *Mean (SD)*.

** *P < 0.05*.

# *Chi-square (and Fisher's exact) test*.

Among the 33 cases under study, 12 (12/33; 36.36%) had only fever, six (6/33; 18.18%) had a dry cough, two (2/33; 6.06%) had vomiting and diarrhea, and 13 (13/33; 39.39%) were placed in the “Others” group, showing symptoms like rhinorrhea, sneezing, sore throat, fatigue, and herpes (Table 2).

**Table 2:** Signs and symptoms of children patients infected with SARS-COV-2.

**No. (%)**	**Total (*n* = 33)**	**Fever (*n* = 21)**	**Non-fever (*n* = 12)**	***t/χ*^**2**^**	^**^p-value
Only fever	12	12 (57.14%)	0	8.45	0.04
^#^Dry cough	6	4 (19.05%)	2 (16.67%)	0.036	0.849
^#^Vomiting and diarrhea	2	2 (9.52%)	0	0.12	0.73
Others	13	3 (14.29%)	10 (83.33%)	12.49	<0.01 (0.0004)

** *P < 0.05*.

# *Chi-square (and Fisher's exact) test*.

**Figure 1:**
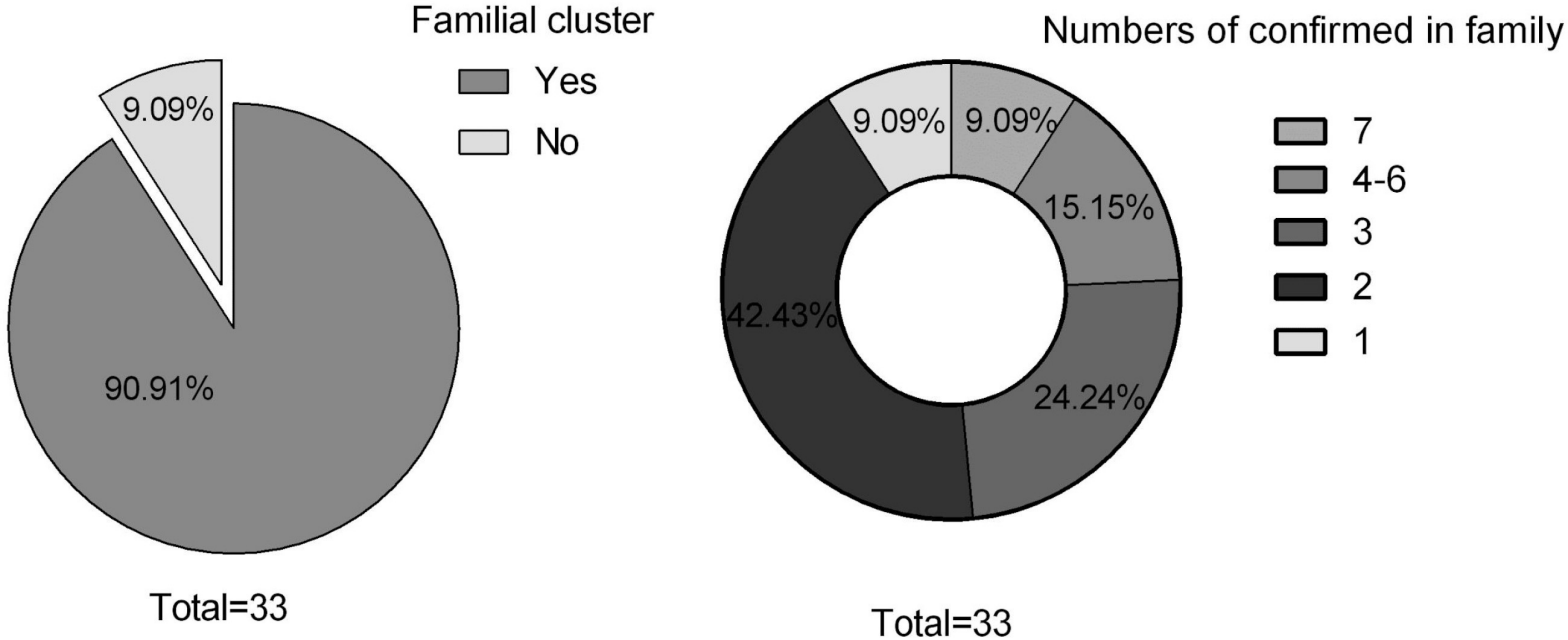
Familial cluster.

**Figure 2:**
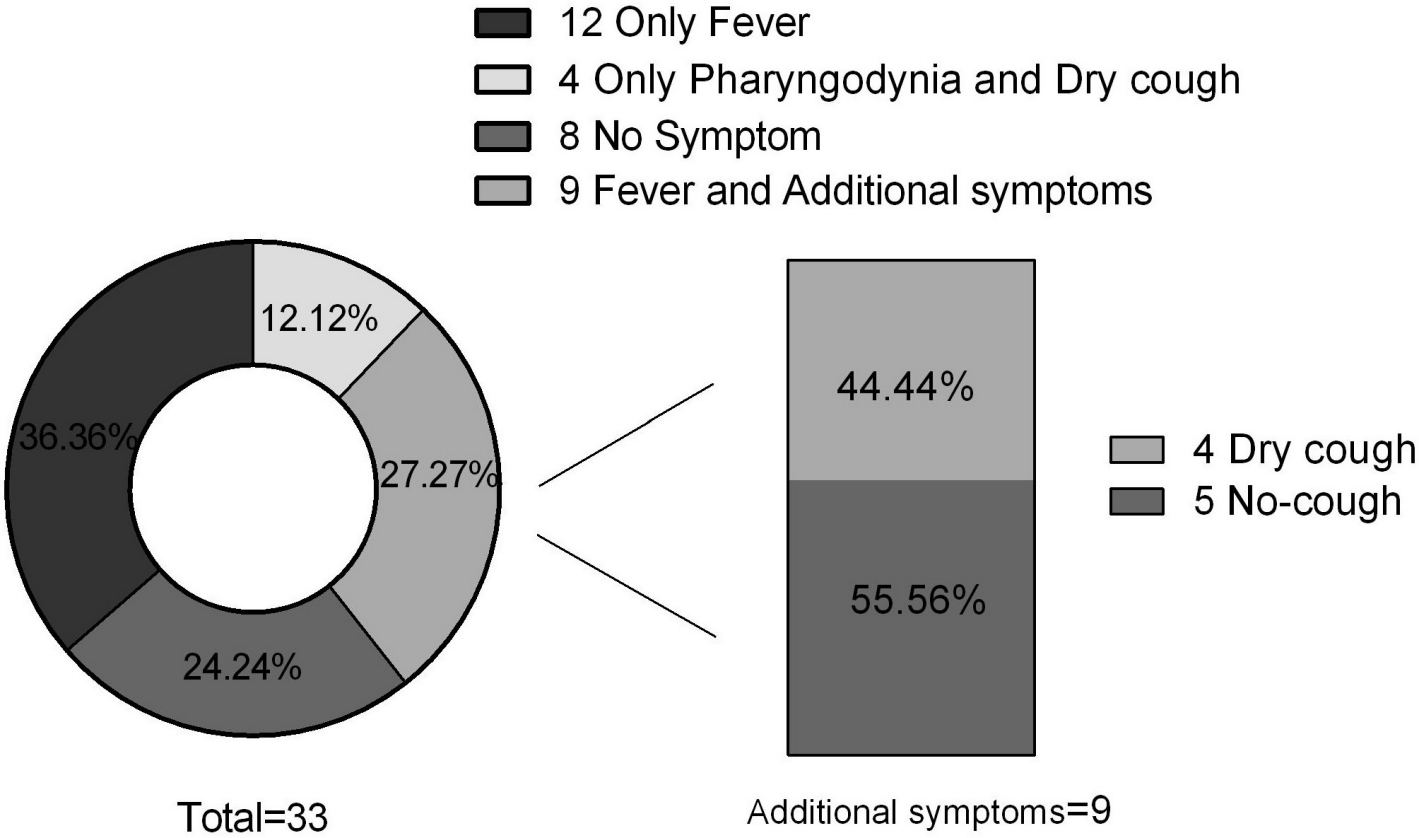
Signs and symptoms.

**Figure 3:**
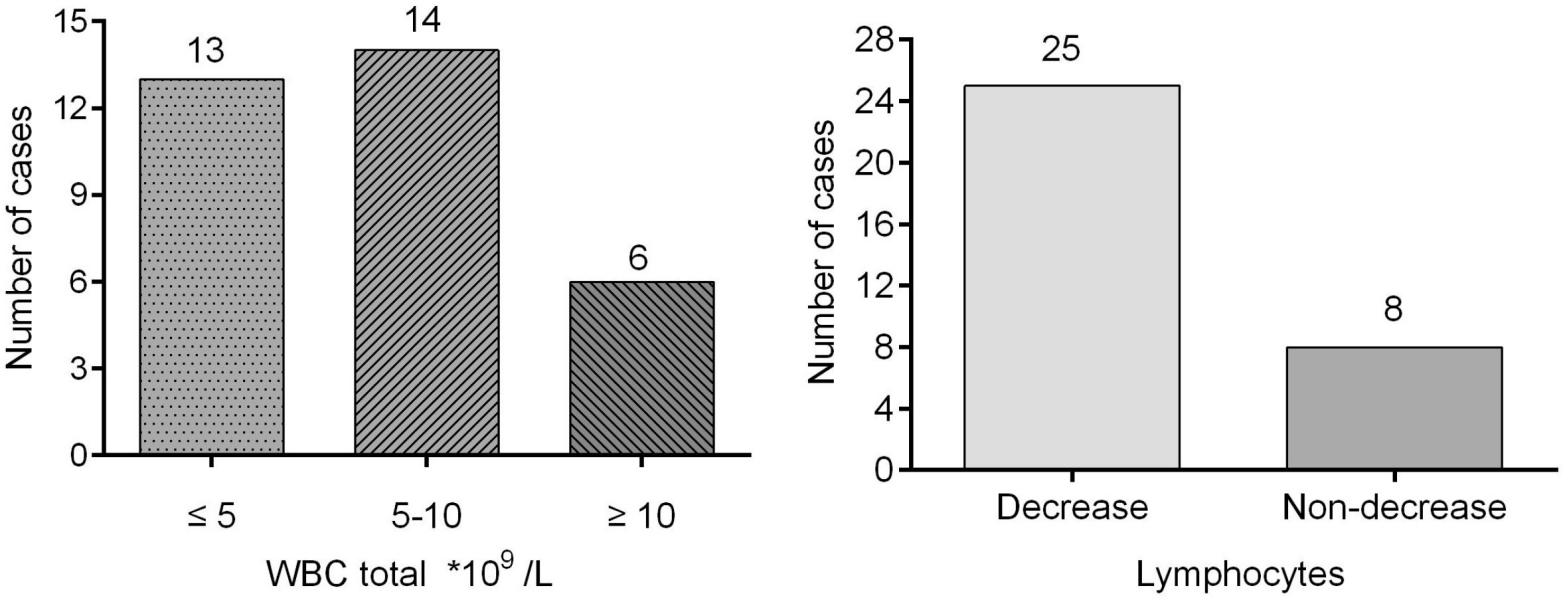
Laboratory test.

**Figure 4:**
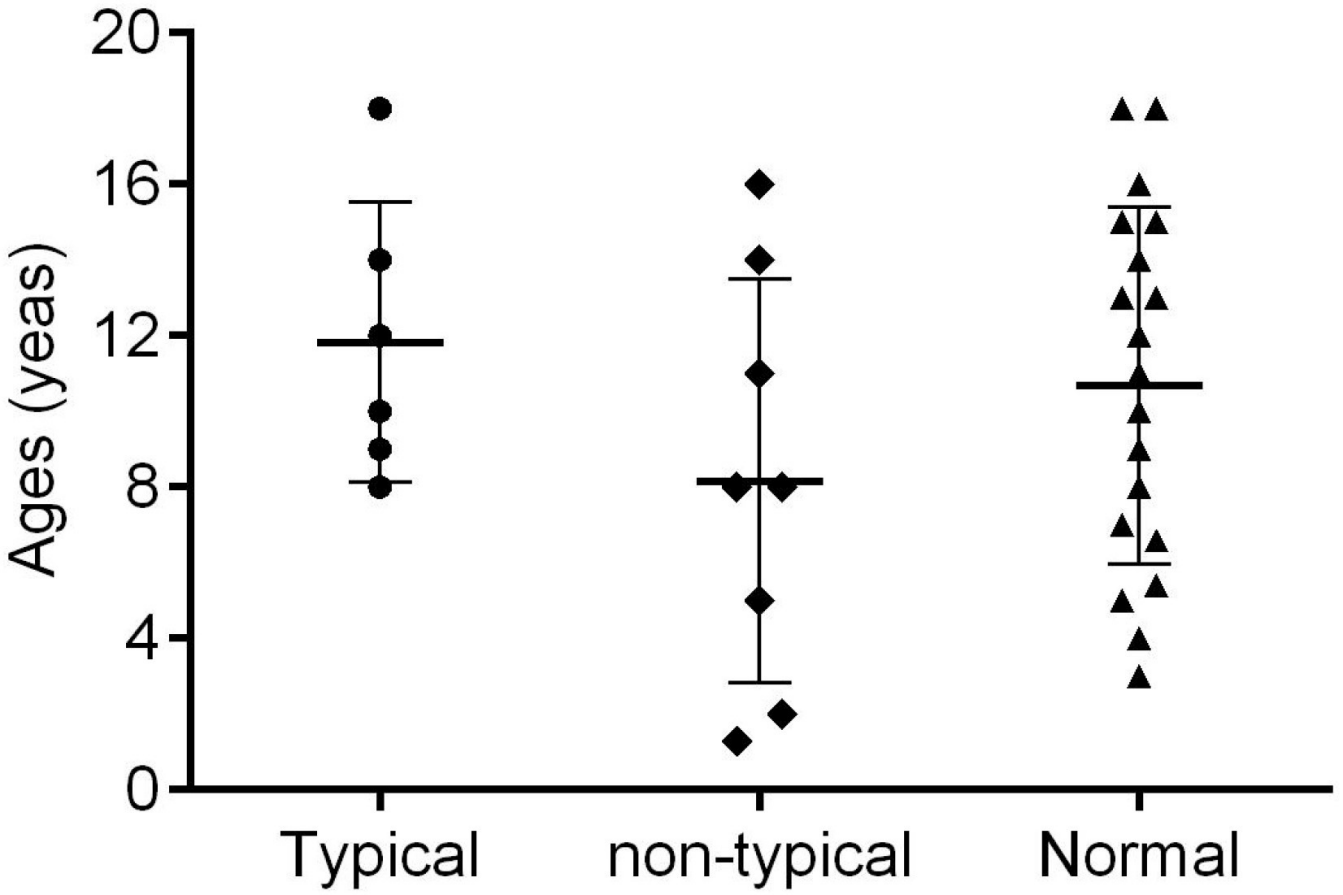
Class of chest CT and age distribution.

Thirty cases (30/33; 90.91%) exhibited familial clustering. There were three families, each of whom had seven members with confirmed SARS-COV-2. Fourteen families (14/33; 42.43%) had two confirmed members (Figure 1). Overall, eight (8/33; 24.24%) cases had no symptoms, 12 (12/33; 36.36%) had only fever, nine (9/33; 27.27%) had both fever and additional symptoms, while 12 (12/33; 36.36%) were without fever. Dry cough was the most common symptom in addition to fever, and additional symptoms included vomiting, diarrhea, and fatigue (Figure 2). Total WBC count was <5^*^109/L in 13 cases, between 5x109/L and 10x109/L in 14 cases, and more than 10x109/L is six cases. Twenty-five cases presented with a decreased lymphocyte population, while eight cases did not (Figure 3). Among the patients under study, seven were under 6 years, 13 were school-age children, and 13 were older than the school-age children. In the three classes based on chest CT images, typical abnormalities occurred in children older than 8 years (Figure 4).

**Figure 5:**
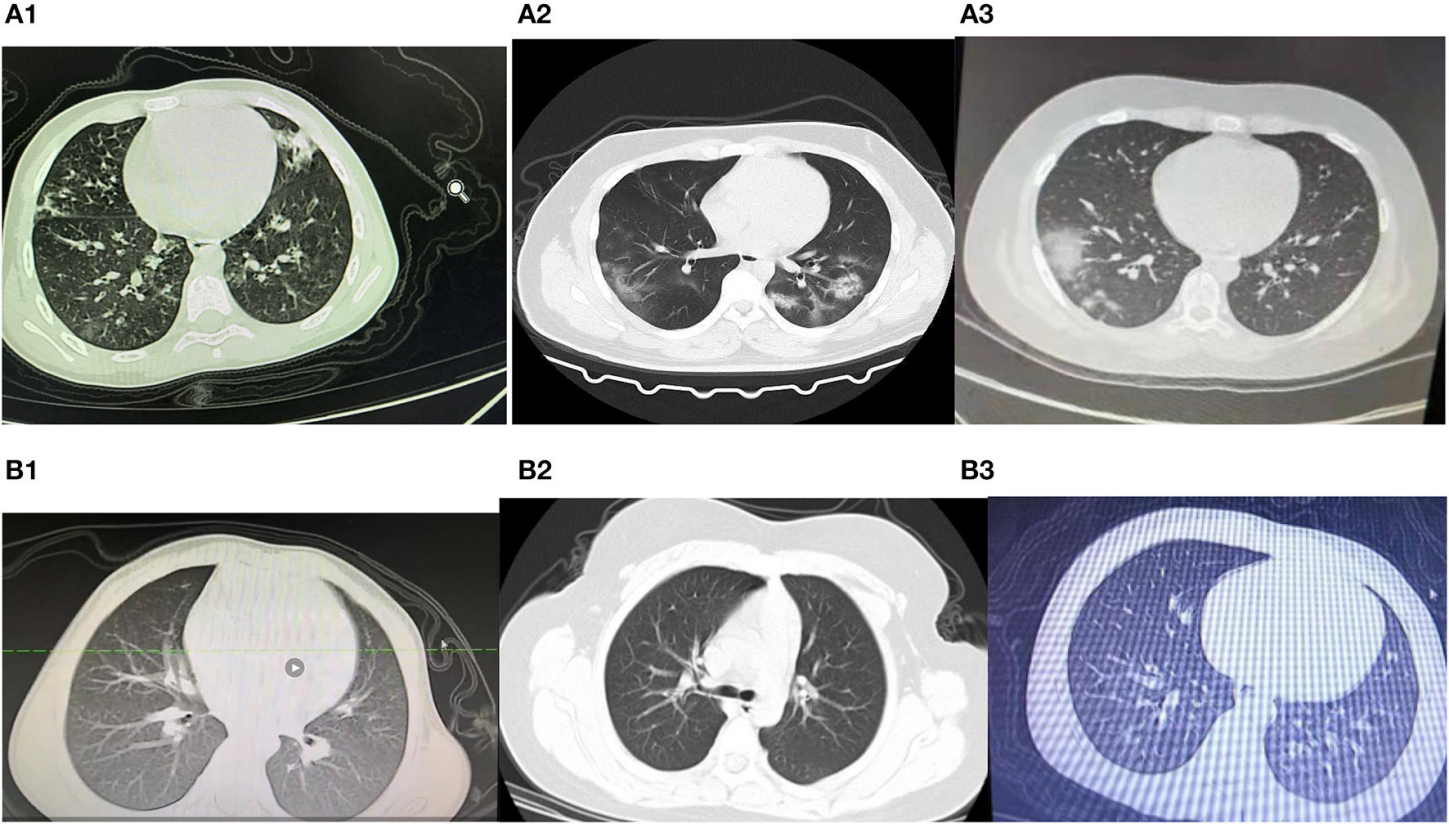
**(A1–3)** Chest CT image with typical abnormalities. **(B1–3)** Chest CT image with non-typical abnormalities.

We divided 33 chest CT images into typical abnormalities (*n* = 6), non-typical abnormalities (*n* = 8), and normal (*n* = 19). The mean age of the group showing typical abnormalities was higher (11.83 ± 3.71 years) than that of the other two groups (8.16 ± 5.32 years and 10.68 ± 4.71 years, respectively), but the differences were not statistically significant. The number of female patients in the normal group was higher than in the other groups and the differences were significant (*P* = 0.038) (Table 3). Most of the typical abnormalities showed GGO with patchy consolidations at subpleural focal changes on CT image (Figure 5A) and non-typical abnormalities CT image mostly showed increased lung marking or dense hilar shadows (Figure 5B).

**Table 3:** Infected with 2019-nCnV.

	**Typical (*n* = 6)**	**Non-typical (*n* = 8)**	**Normal (*n* = 19)**	***t/χ*^**2**^**	^**^p-value
^*^Age	11.83 ± 3.71	8.16 ± 5.32	10.68 ± 4.71	1.200	0.315
^#^Sex					
Female	1	3	13	6.531	0.038
Male	5	5	6		

* *Mean (SD)*.

** *P < 0.05*.

# *Chi-square (and Fisher's exact) test*.

Among various treatment categories, nine cases were administered with only INF-a, 17 cases had INF-a combined with other antiviral drugs, including Lopinavir and Ritonavir, ribavirin, and Arbido, while four cases used only Chinese patent medicine as an antiviral drug. We did not find any difference in the curative effects of these drugs.

## Discussion

We present here a descriptive study on the clinical and epidemiological characteristics of the COVID-19 infection. We collected data on 33 young patients (<19 years of age) who were admitted to one of the 10 hospitals in Anhui Province, China. This report presents the latest data and status of COVID-19 infection in Anhui Province, China.

As of February 23, 77048 laboratory-confirmed SARS-CoV-2 infections were reported in China. Among these, 2.1% (3) were below 19 years of age. Coronavirus is an enveloped, positive-sense, single-stranded RNA virus, capable of rapid mutations and recombination. This novelβ-coronavirus belongs to lineage B or subgenus sarbecovirus, that includes the human SARS coronavirus (4).

As of February 23, 2020, among the 33 patients included in this study, no dyspnea or similar complications were reported, and none of them were critically ill.

COVID-19 infection is associated with clustering onset (5). The data in this cohort study showed that only three patients had no familial clustering history, including two patients who were residents of Wuhan. A majority of these patients (30, or 30/33; 90.91%) cases showed familial clustering. Three families had seven members each, and five families had four patients each with confirmed infection. Among all cases, two were twins, two were sisters, and four were cousins. This suggests rapid person-to-person transmission of COVID-19, similar to what happens in adults. COVID-19 is mainly transmitted through respiratory droplets or through contact (6). In addition, current research shows that it may also be transmitted through the fecal-oral route (7), inhalation through aerosols produced through coughing by the infected family members, relatives, and healthcare workers, or though other sources in the environment (8). A recent study also suggested that infection in the womb or during birth could not be denied completely (9). Nevertheless, in children, familial clustering is an important factor in rapid human-to-human transmission of COVID-19 through close family contacts. Therefore, vigilant control measures should be taken at an early stage of the infection in a family (4).

As other studies reported, we noticed that SARS-COV-2 less commonly affects children (10), and that they have much fewer symptoms and less severe cases (11) compared with adults, and also much lower case-fatality rates (10). In our study, none of the cases had difficulty breathing or needed oxygen support; this is different to adult cases. The common symptoms at the onset of illness were fever and dry cough. Huang (12) reported fever [40 of 41 patients [98%]], cough (76%), myalgia or fatigue (44%), headache (8%), hemoptysis (5%), and diarrhea (3%). Wang et al. (13) reported common symptoms, including fever (98.6%), fatigue (69.6%), dry cough (59.4%), myalgia (34.8%), and diarrhea and nausea (10.1%). However, 12 (12/33; 36.36%) cases in the present study were without fever and a small proportion of patients presented initially with atypical symptoms, like fatigue, sore throat, rhinorrhea, sneezing, vomiting, diarrhea, and herpes. One of them had a sore throat at the onset of symptoms and one had fatigue; the status was the same as that of adults. A 27-year-old man (14) was reported with vomiting and loose stools before admission. Michelle et al. (15) reported the first case in the United States which was that of a 35-year-old man, with a “subjective fever” of 37.2°C. This patient presented with a persistent dry cough, nausea, and vomiting. In a report (5) of 99 cases, 20% had no fever or cough at the onset. This suggests that measuring the body temperature cannot be considered as a decisive screening method. Furthermore, in our report, there were eight (8/33; 24.24%) cases without any early signs or symptoms. When present, the signs and symptoms were from the respiratory system (upper and lower) to the digestive system. We speculate that this observation probably indicates that the target cells might be located in different tissues, and this may change with age.

In most of the cases enrolled in this study, the total WBC count was normal or decreased. The percentage of lymphocytes decreased in 25 (25/33; 75.75%) cases. Many reports (12, 13, 15, 16) of adults showed the routine blood test was useful as a diagnostic tool. A decrease in lymphocyte count indicates that SARS-COV-2 affects immune cells and inhibits cellular immune function (5). T lymphocyte damage (17) might be an important factor in exacerbating the condition of patients. The decreasing percentage of lymphocytes could prompt SARS- COV-2 infections in the clinic. In addition, Huang et al. (12) reported that 40% of the cases they studied showed hepatic injury, five cases had myocardial injury, and injuries were more severe in critical patients. In our study, none of the patients showed myocardial injury, only two 18-year-old patients showed hepatic involvement. This difference may be attributed to better liver regeneration capacity and better ability to recover from myocardial injury.

Six (6/33; 18.18%) cases had typical GGO or consolidation (18) of the lungs as the primary findings on CT scans. All the patients were more than 8 years old. The infants and preschool-age children had atypical chest CT scans or normal CT. A familial clustering report suggested that the symptoms of COVID-19 were non-specific, but the three oldest patients in that family had more critical symptoms (4). It may be because the trachea, bronchi, and capillaries are relatively thin in childhood, and children's lungs are rich in connective tissue, poorly developed elastic tissue, abundant blood vessels, capable of holding less air, have fewer alveoli, and a less well-developed pulmonary interstitium. More research focused on the function of ACE2 as the SARS-CoV-2 receptor and proved the binding of SARS-CoV-2 to ACE2 lead to driving the systemic manifestations of COVID-19, including respiratory clinical feature and cardiovascular complication (19). Most elderly patients routinely take ACE2 receptor antagonists to treat high blood pressure, which increases the expression of ACE2 and helps COVID-19 enter the cells. On the contrary, the level of ACE2 expression in children is low and therefore the symptoms are mild. In both healthy and diabetic individuals, ACE2/ACE is negatively correlated with age (20). Many reports have shown that older males (21–24) are more likely to be infected by COVID-19. The atypical and normal chest CT scans suggest that more attention needs to be paid to young children. We also observed a greater number of males than females with typical CT scans. In adults (5), the proportion of confirmed infection in men is higher than in women. However, Wei et al. (25) reported nine infected infants from 1 to 11 months, and seven of them were females. Thus, COVID-19 is more likely to infect adult and older males (21–24).

On January 9, 2020, Chinese scientists identified the cause of a new illness as a novel coronavirus, and as of January 10, 41 confirmed cases of coronavirus pneumonia had been reported in Wuhan city. This is the first time this disease was called “NCP.” This new virus was designated as WH-Human 1 coronavirus (WHCV) (26) and has also been referred to as “2019-nCoV.” Huang et al. (12) reported that all patients had pneumonia. The virus was given the official name of COVID-19 by the WHO on February 11, 2020 (27), and this name is more scientific and suitable. In this retrospective study, we report 12 cases without fever and eight cases without any signs and symptoms, and all cases were mild. Only six cases had typical GGO or consolidation on CT scans. We divided the patients into those with typical signs and symptoms such as fever, dry cough, and atypical sore throat, fatigue, vomiting, and diarrhea. We also divided the chest CT scans into typical, atypical, and normal. We suggest more attention should be paid on the children without syndrome but with family member infected by COVID-19.

This is a small case report of patients admitted to different hospitals, and the test results and chest CT scan results were not homogenous. It is necessary to follow up the cases enrolled in this study until all of them are discharged from the hospital, and also to test the respiratory specimens 2 weeks after discharge to re-confirm that all of them are cured of SARS-COV-2 infection.

## Conclusion

Children's infection is mild and familial clustering was the most common channel of infection. The older patients had more typical ground glass opacity (GGO) or consolidation in chest CT scans. Cases without fever strongly suggested that non-symptomatic children should not be assumed to be free of infection when their family members have confirmed infection. Children were highly susceptible to COVID-19 and they showed clinical features distinguishbale from adults.

## Data Availability Statement

All datasets generated for this study are included in the article/supplementary material.

## Ethics Statement

The studies involving human participants were reviewed and approved by Anhui Provincial Hospital Medical Research Ethics Committee. Written informed consent to participate in this study was provided by the participants' legal guardian/next of kin. Written informed consent was obtained from the individual(s) for the publication of any potentially identifiable images or data included in this article.

## Author Contributions

LZ and SH contributed to the conception and design of research. LZ gathered the medical data, updated the literature search, made independent quality assessments, and extracted data before comparing results and resolving differences. The NICU team of the First hospital affiliated to USTC analyzed the data. LZ edited and revised the manuscript. SH and LZ approved the final version of manuscript.

## Conflict of Interest

The authors declare that the research was conducted in the absence of any commercial or financial relationships that could be construed as a potential conflict of interest.
